# Stochastic platform based on calix[6]arene and TiO_2_-modified reduced graphene oxide electrode for on-site determination of nonivamide in pharmaceutical and water samples

**DOI:** 10.1039/d3ra02363j

**Published:** 2023-06-12

**Authors:** Bianca-Maria Tuchiu, Raluca-Ioana Stefan-van Staden, Jacobus (Koos) Frederick van Staden

**Affiliations:** a Laboratory of Electrochemistry and PATLAB, National Institute of Research for Electrochemistry and Condensed Matter 202 Splaiul Independentei Str. 060021 Bucharest-6 Romania ralucavanstaden@gmail.com +40751507779; b Faculty of Chemical Engineering and Biotechnologies, Politehnica University of Bucharest Bucharest Romania

## Abstract

Using a detection platform based on an integrated sensor constructed by modifying TiO_2_ and reduced graphene oxide paste with calix[6]arene, a novel stochastic approach for both quantitative and qualitative analysis of nonivamide in pharmaceuticals and water samples has been developed. A wide analytical range of 1.00 × 10^−18^ to 1.00 × 10^−1^ mol L^−1^ was obtained with the stochastic detection platform for nonivamide determination. A very low limit of quantification of 1.00 × 10^−18^ mol L^−1^ was reached for this analyte. The platform was successfully tested on real samples, respectively, on topical pharmaceutical dosage form and surface water samples. The samples were analyzed without pretreatment in the case of pharmaceutical ointment or under minimal preliminary processing for surface waters proving a facile, rapid, and reliable method. Moreover, being portable, the developed detection platform is adequate for on-site analysis in various sample matrices.

## Abstract 2

Calix[6]arene recognised nonivamide in topical ointments and in water samples.

## Graphical abstract

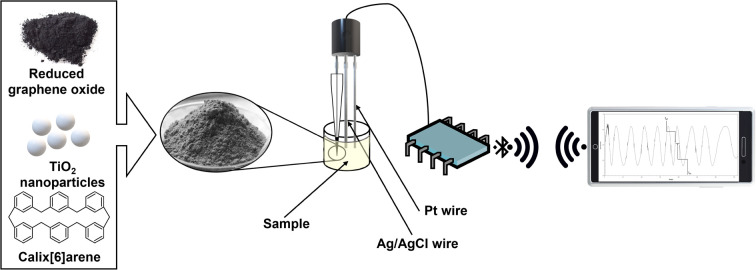

## Introduction

**Fig. 1:**
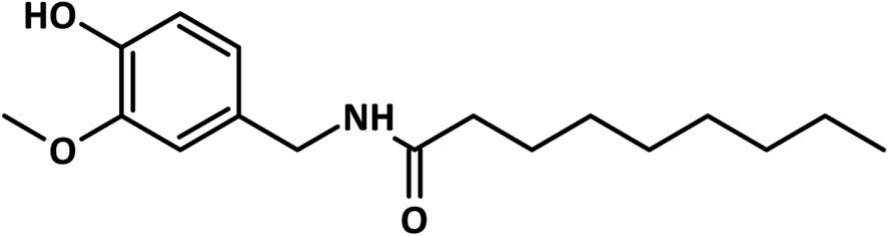
The chemical structure of nonivamide.

Nonivamide (Fig. 1) is a capsaicin analogue that naturally occurs in *Capsicum oleoresin*. Similar to other compounds from the capsaicin class, nonivamide is used as a food additive to confer pungency to foods, as an active ingredient in various anti-inflammatory and analgesic topical formulations, and as an anti-riot agent due to its lachrymatory effect.^1,2^ Moreover, it has been proven that it can be used as a marine antifouling paint, which prevents various marine organisms from adhering to or growing on underwater surfaces.^3,4^ Recently, nonivamide has been shown to have anti-obesity properties by regulating lipid metabolism and satiety, making it suitable for use as a dietary supplement in maintaining normal body weight.^5,6^

When considering the adverse effects of capsaicinoids, such as severe cough, transient spasms of the respiratory tract, and irritation of the eyes, skin, and mucous membranes, their use should be properly managed since they may lead to significant impairment in the functions of the human body.

To date, several methods have been reported for the determination of nonivamide, these being liquid chromatography-tandem mass spectrometry (LC-MS/MS), ultra-high performance liquid chromatography–tandem mass spectrometry (UPLC–MS/MS), and UPLC with UV detection.^7–9^ While these techniques are reliable, repeatable, and sensitive, they possess certain disadvantages such as expensive instruments and reagents, time-consuming sample pretreatment, and the necessity of trained professionals. Since electrochemical methods are sensitive, selective, easier to use, inexpensive, and require minimal sample processing, they may be a suitable alternative for the determination of nonivamide. Only one electrochemical sensor has been developed for this purpose.^10^

The stochastic sensor-based determination approach allows both qualitative and quantitative analysis. To date, many stochastic sensors have been proposed for screening certain diseases, assessing water quality, and determining certain pharmaceutically active ingredients.^11–14^

In this work, we propose the use of a portable platform based on a sensor obtained by modifying a matrix comprising TiO_2_ and reduced graphene oxide paste with calix[6]arene (C6A/TiO_2_/rGOPE) for the on-site qualitative and quantitative determination of nonivamide in real samples (topical pharmaceutical dosage form and surface water). Calix[6]arene was chosen as a material in the sensor design because it presents the cavity (channel) of 5 Å (ref. 15) needed for producing the stochastic signal. Reduced graphene oxide has excellent conductive and electrical properties, similar to those of pristine graphene due to its heterogeneous structure composed of basal planes of hexagonally displayed sp^2^ hybridized carbon atoms. As a result, it is often utilized in the development of electrochemical sensors.^16,17^ TiO_2_ is an inexpensive, stable, non-toxic, electrically conductive material capable of improving both sensor response and stability.^18^ The novelty of the work is given by the utilization of a C6A/TiO_2_/rGOPE-based sensor as part of a portable platform for on-site analysis of nonivamide in pharmaceutical compounds and water samples.

## Experimental

### Materials and reagents

Nonivamide, titanium(iv) oxide nanopowder, calix[6]arene, monosodium phosphate, disodium phosphate, dimethyl sulfoxide, and chloroform were procured from Sigma Aldrich (Milwaukee, USA). The paraffin oil (d_4_^20^, 0.86 g cm^–1^) was procured from Fluka (Buchs, Sweden). Graphene oxide powder was procured from NannoInnova Technologies (Toledo, Spain).

By mixing monosodium phosphate and disodium phosphate aqueous solutions, a phosphate buffer solution (PBS, 0.10 mol L^–1^) was prepared. Using a 0.10 mol L^–1^ HCl solution, the pH of the buffer solution was adjusted to the desired pH of 5.00. Nonivamide was dissolved in dimethyl sulfoxide to prepare the stock solution (1.00 × 10^–2^ mol L^–1^).

A 10^−3^ mol L^−1^ solution of calix[6]arene was obtained by its dissolution in dimethyl sulfoxide.

### Apparatus and methods

An EmStat Pico mini potentiostat coupled to a smartphone with PStouch mobile application version 2.7 (PalmSens BV, Houten, the Netherlands) was used for all stochastic measurements. In a conventional three-electrode electrochemical cell, the working, reference, and counter electrodes were C6A/TiO_2_/rGOPE, an Ag/AgCl wire (1.00 mol L^–1^ KCl), and a Pt wire.

A Mettler Toledo pH meter was used to adjust the pH of the solutions. For the preparation of the solutions, deionized water was obtained from a Direct-Q 3 Water Purification System (Molsheim, France).

The measurements were conducted at room temperature.

### Design of the C6A/TiO_2_/rGOPE platform

For the preparation of the sensor, a physical mixture of 100 mg reduced graphene oxide, 10 mg TiO_2_, and an adequate quantity of paraffin oil was modified with 100 μL calix[6]arene solution (10^−3^ mol L^−1^). The sensor was assembled by placing the paste in a non-conducting plastic tube and electrically connecting it to the external circuit with a silver wire. The platform was assembled by integrating the sensor into the electrochemical cell and connecting it to the mini potentiostat. The surface of the electrode was renewed by polishing it with aluminum foil. Before each measurement, the sensor was cleaned with deionized water to remove any contaminants. C6A/TiO_2_/rGOPE was stored at room temperature, in a dry place.

### Stochastic mode

The stochastic mode was used for all measurements. Channel conductivity is the underlying principle of stochastic sensing. *t*_off_ and *t*_on_ parameters were identified, the former represents the signature of the analyte, while the latter's value was used for the quantitative analysis of the analyte. The *t*_on_ and *t*_off_ values were determined using the chronoamperometry technique. The current was measured after applying a 200 mV *vs.* Ag/AgCl constant potential. The calibration of the platform was conducted using standard solutions with different amounts of nonivamide. The signature of nonivamide (*t*_off_ parameter) identified in the diagrams was employed for the qualitative analysis while the *t*_on_ parameter was used for the quantitative analysis, according to the linear dependency:1/*t*_on_ = *a* + *b* × *C*_nonivamide_where *a* – intercept, and *b* – slope/sensitivity.

The linear regression approach was applied to establish the calibration equation. The unknown concentrations of nonivamide in samples were calculated based on this equation.

### Samples

The applicability of the proposed platform was tested on real samples: pharmaceutical ointment and surface water samples.

The pharmaceutical ointment was procured from a local drug store. It contained 4 mg per gram of nonivamide and 25 mg per gram of nicoboxil as active pharmaceutical ingredients. The ointment also contained excipients such as diisopropyl adipate, colloidal silicon dioxide, white vaseline, sorbic acid, ceylon citronella oil, and purified water. No preliminary processing of the samples was performed.

Surface water samples were taken from a local river located and kept in the refrigerator until examination. pH 5.00 PBS was used to buffer the samples, in the ratio of 1 : 1 (v/v). No nonivamide signature was identified in the buffered samples, indicating that the samples did not contain this molecule. For this reason, the samples were spiked with several known concentrations of nonivamide.

## Results and discussion

### Response characteristics of the sensor in the stochastic mode

Stochastic detection is a two-step process. In the first step, the current flows through the channel at an applied potential of 200 mV and is modified by the extraction of the analyte from the solution at the membrane–solution interface, blocking the channel and causing the current to drop to 0. This step is called pattern recognition and the period of time during which it occurs defines the analyte signature (*t*_off_ parameter) and provides information on the qualitative analysis. The analyte signature, and therefore, the time in which the first step takes place is dependent on the size, geometry, and, in the case of proteins, the rate at which they unfold. In the second phase, called the binding phase, the analyte enters the channel where it binds to the channel wall and undergoes redox processes according to the following equilibrium equation:Ch_(i)_ + nonivamide_(i)_ ⇔ Ch·nonivamide_(i)_where Ch – is the channel and i – the interface.

The duration of the second stage represents the *t*_on_ parameter that provides useful information for quantitative analysis.

In Table 1, the response characteristics of the C6A/TiO_2_/rGOPE platform obtained from the values of the *t*_on_ parameter are presented. By correlating the wide linear range with the very low quantification limit and high sensitivity obtained, it can be concluded that the proposed platform represents a reliable method for the determination of nonivamide in pharmaceutical and water samples with minimal sample processing. Hence, the proposed method can be applied in the quality control of topical pharmaceutical dosage forms as well as in the on-site monitoring of surface water quality.

**Table 1:** Response characteristics of the C6A/TiO_2_/rGOPE platform

Calibration equation; correlation coefficient (*r*)	Linear concentration range (mol L^–1^)	*t*_off_ (s)	Sensitivity (s^–1^ mol L^–1^)	LOQ (mol L^–1^)
1/*t*_on_ = 0.46 + 9.76 × 10^2^ × *C*_nonivamide_, *r* = 0.9980	1.00 × 10^−18^ to 1.00 × 10^−1^	0.4	9.76 × 10^2^	1.00 × 10^−18^

The proposed platform demonstrated a very low limit of quantification (LOQ) and a larger linear concentration range when compared with previous methods reported for nonivamide determination. The comparison is presented in Table 2.

**Table 2:** Comparison of different proposed methods used for nonivamide determination

Method	Linear concentration range (mol L^–1^)	LOQ (mol L^–1^)	Ref.
LC–MS/MS	3.40 × 10^–9^ to 8.52 × 10^–7^	3.40 × 10^–9^	7
UPLC–MS/MS	1.70 × 10^–10^ to 1.36 × 10^–8^	1.70 × 10^–10^	8
UPLC with UV detection	3.41 × 10^–6^ to 3.41 × 10^–4^	6.48 × 10^–7^	9
PGA/MWNT/GCE^a^	2.50 × 10^–8^ to 7.50 × 10^–5^	2.00 × 10^–8^	10
Stochastic platform	1.00 × 10^−18^ to 1.00 × 10^−1^	1.00 × 10^−18^	This work

a PGA/MWNT/GCEa – glassy carbon electrode modified with multi-walled carbon nanotubes and poly(gallic acid).

The stability of the proposed stochastic platform was checked in time. The platform was tested for a period of 6 months, and the sensitivity was measured every day during this period of time. The variation of the sensitivity was 0.73% at the end of the six months period of measurements, proving its high stability in time. Also, high recoveries were recorded when used for on-site continuous assay of nonivamide in surface water samples for a period of one month. The platform was also used continuously to assess the uniformity content of nonivamide in topical pharmaceutical dosages in a specialized laboratory when, after one month, the accuracy of measurements *vs.* HPLC methods was as high as 99.00%.

The selectivity of the proposed stochastic platform was checked *versus* capsaicin (a pharmaceutical compound with a similar structure), diisopropyl adipate, colloidal silicon dioxide, and sorbic acid (used as excipients in the formulation of topical pharmaceutical dosages). The signatures obtained for these substances were far higher than 0.4 s proving that nonivamide can be determined selectively in their presence in either water or pharmaceutical formulations. To date, we did not find any interferent (a compound that may have the same signature), although we tested several similar compounds that may be found as by-products or in surface water, and therefore there may be interferences related to other unknown compounds, to date.

### Determination of nonivamide from a topical pharmaceutical dosage form and surface water samples

**Fig. 2:**
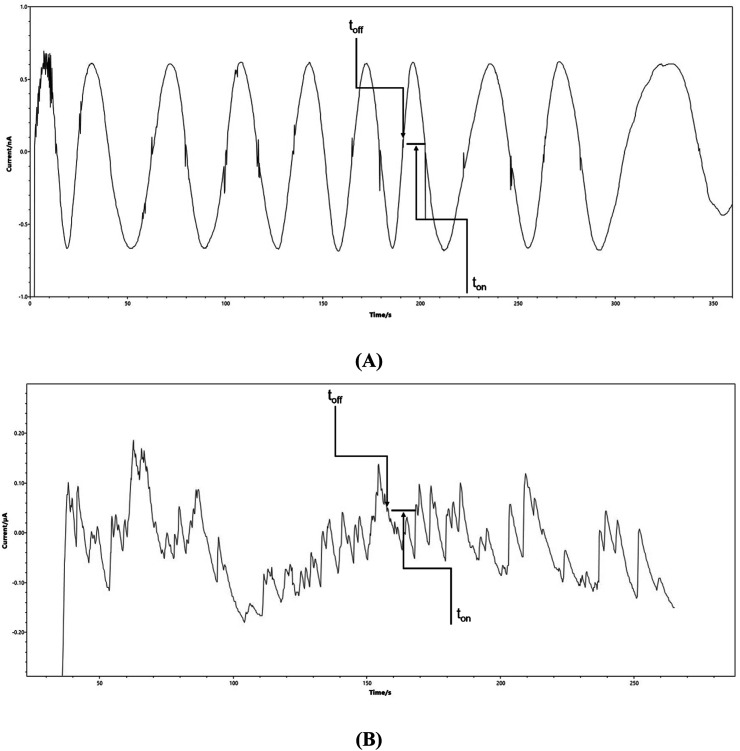
Stochastic diagrams recorded using the C6A/TiO_2_/rGOPE platform for the determination of nonivamide in (A) topical pharmaceutical dosage form, and (B) surface water samples.

The proposed detection platform was used for the determination of nonivamide in topical pharmaceutical dosage and surface water samples. After the measurements, the nonivamide signature (*t*_off_) together with the corresponding *t*_on_ value were identified and read off the recorded diagrams, as depicted in Fig. 2. The nonivamide concentrations in the samples were determined using the obtained calibration curve. The calculated recovery and RSD values are summarized in Table 3.

**Table 3:** Determination of nonivamide in a topical pharmaceutical dosage form sample and spiked surface water samples using the proposed C6A/TiO_2_/rGOPE platform (*N* = 10)

Sample	Nonivamide added amount (mol L^−1^)	Recovery (%)	RSD (%)
Topical pharmaceutical dosage form sample	—	92.50	0.03
Surface water samples	10^−16^	96.28	0.04
	10^−13^	96.97	0.03
	10^−9^	94.43	0.02
	10^−4^	99.98	0.03

According to the results, the proposed platform is highly reliable for the qualitative and quantitative assay of nonivamide in real samples and can be used for on-site analysis of pharmaceutical products and waters. In addition, the use of the portable platform with the stochastic method, which requires minimal pretreatment of the samples, makes it suitable for on-site analysis in both drug quality control and surface water quality screening.

Utilization of stochastic mode facilitates the qualitative and quantitative analysis in very complex matrices because the analysis takes place reliably in two phases: recognition phase – based on its signature, and determination phase – based on measurement of the time measured in between two consecutive signatures, this quantification taking place inside the channel in a closed environment.

## Conclusions

The proposed stochastic platform can be reliably used for the on-site control of the purity of nonivamide during the synthesis process, for the on-site control of the uniformity content test of the ointment containing nonivamide, and also for the on-site control of the quality of water samples. The main feature is its utilization on a large scale for continuous assay of water quality and also implemented by the pharmaceutical industry for on-site purity control of the raw substance.

## Conflicts of interest

There are no conflicts to declare.

## Supplementary Material
